# Supplementary material for: Indications of Induction and Caesarean Sections Performed Using the Robson Classification in a University Hospital in Spain from 2010 to 2021
Source: Healthcare (Basel). 2023 May 23;11(11):1521. doi: 10.3390/healthcare11111521 (PMC10252359; doi:10.3390/healthcare11111521)
Supplement: Supplementary file 1 [file healthcare-11-01521-s001.zip › healthcare-2308891-supplementary.pdf]

Supplementary Table S1. Distribution of socio-demographic and obstetric variables of the different RTGCS from 2010 to 2021 in the HULR.

Group 1		2010-2013		2014-2017		2018-2021		
		n	*%*	*n*	*%*	*n*	*%*	*p*-value*
Country of origin	Spain	2079	81.2%	1885	78.8%	1146	60.6%	<0.001
	Foreign	480	18.8%	507	21.2%	745	39.4%	
Newborn sex	Male	1300	50.8%	1223	51.1%	951	50.3%	0.886
	Female	1260	49.2%	1169	48.9%	940	49.7%	
			Period		*n*	Mean	*SD*	*p*-value**
	Maternal age		2010-2013		2559	30.2	5.7	0.005
			2014-2017		2392	29.9	5.9	
			2018-2021		1891	29.6	6.2	
	Birth weight		2010-2013		2559	3303.6	413.8	0.081
			2014-2017		2392	3285.5	405.4	
			2018-2021		1891	3314.9	411.3	

Group 2		2010-2013		2014-2017		2018-2021		
		n	*%*	*n*	*%*	*n*	*%*	*p*-value*
Country of origin	Spain	1056	81.9%	881	82.3%	621	68.0%	<0.001
	Foreign	233	18.1%	189	17.7%	292	32.0%	
Newborn sex	Male	651	50.5%	546	51.0%	473	51.8%	0.839
	Female	638	49.5%	524	49.0%	440	48.2%	
			Period		*n*	Mean	*SD*	*p*-value**
	Maternal age		2010-2013		1289	30.7	5.3	0.012
			2014-2017		1070	31.1	5.8	
			2018-2021		913	31.2	6.1	
	Birth weight		2010-2013		1289	3285.6	477.3	0.431

		Period	n	Mean	SD	
		2014-2017	1070	3269.1	493.1	
		2018-2021	913	3284.4	516.7	

Group 3		2010-2013		2014-2017		2018-2021		
		n	*%*	*n*	*%*	*n*	*%*	*p*-value*
Country of origin	Spain	2133	82.9%	1796	83.6%	1333	78.2%	<0.001
	Foreign	440	17.1%	353	16.4%	372	21.8%	
Newborn sex	Male	1332	51.8%	1093	50.9%	890	52.2%	0.737
	Female	1241	48.2%	1056	49.1%	815	47.8%	

	Period	*n*	Mean	*SD*	*p*-value**
Maternal age	2010-2013	2573	30.0	5.4	<0.001
	2014-2017	2149	31.7	5.4	
	2018-2021	1705	31.9	5.4	
Birth weight	2010-2013	2573	3336.2	404.5	<0.001
	2014-2017	2149	3387.3	416.5	
	2018-2021	1705	3387.0	405.1	

Group 4		2010-2013		2014-2017		2018-2021		
		n	*%*	*n*	*%*	*n*	*%*	*p*-value*
Country of origin	Spain	837	86.0%	407	82.2%	301	83.1%	0.056
	Foreign	136	14.0%	88	17.8%	61	16.9%	
Newborn sex	Male	508	52.2%	252	50.9%	178	49.2%	0.786
	Female	465	47.8%	243	49.1%	184	50.8%	

	Period	*n*	Mean	*SD*	*p*-value**
Maternal age	2010-2013	973	30.6	4.8	<0.001
	2014-2017	495	32.3	5.2	

		2018-2021		362	32.7	5.3	
Birth weight		2010-2013		973	3355.8	469.5	0.617
		2014-2017		495	3361.6	477.7	
		2018-2021		362	3383.4	530.2	

Group 5		2010-2013		2014-2017		2018-2021		
		n	*%*	*n*	*%*	*n*	*%*	*p*-value*
Country of origin	Spain	104	81.3%	70	76.9%	79	80.6%	0.684
	Foreign	24	18.8%	21	23.1%	19	19.4%	
Newborn sex	Male	73	57.0%	53	58.2%	54	55.1%	0.977
	Female	55	43.0%	38	41.8%	44	44.9%	

	Period	*n*	Mean	*SD*	*p*-value**
Maternal age	2010-2013	128	31.1	5.3	<0.001
	2014-2017	91	33.5	4.9	
	2018-2021	98	35.0	4.9	
Birth weight	2010-2013	128	3464.3	521.4	0.747
	2014-2017	91	3526.0	573.5	
	2018-2021	98	3475.9	530.6	

Group 6		2010-2013		2014-2017		2018-2021		
		n	*%*	*n*	*%*	*n*	*%*	*p*-value*
Country of origin	Spain	144	90.6%	118	88.1%	85	78.0%	<0.001
	Foreign	15	9.4%	16	11.9%	24	22.0%	
Newborn sex	Male	71	46.4%	71	53.4%	56	48.3%	0.275
	Female	82	53.6%	62	46.6%	60	51.7%	

	Period	*n*	Mean	*SD*	*p*-value**

	Maternal age	2010-2013		159	31.5	5.5	0.817
		2014-2017		134	31.4	5.5	
		2018-2021		109	31.2	6.4	
	Birth weight	2010-2013		159	3138.5	460.9	0.689
		2014-2017		134	3113.2	481.6	
		2018-2021		109	3149.3	530.0	
**Group 7**		**2010-2013**		**2014-2017**		**2018-2021**	
		n	**%**	***n***	**%**	***n***	**%**	***p*-value***
Country of origin	Spain	77	86.5%	41	82.0%	32	80.0%	0.689
	Foreign	12	13.5%	9	18.0%	8	20.0%	
Newborn sex	Male	45	50.6%	20	40.0%	18	45.0%	0.489
	Female	44	49.4%	30	60.0%	22	55.0%	
		**Period**		***n***	**Mean**	***SD***	***p*-value****
	Maternal age	2010-2013		89	31.6	5.2	<0.001
		2014-2017		50	32.9	5.6	
		2018-2021		40	35.1	4.1	
	Birth weight	2010-2013		89	3207.4	533.7	0.929
		2014-2017		50	3152.6	498.6	
		2018-2021		40	3227.9	470.1	
**Group 8**		**2010-2013**		**2014-2017**		**2018-2021**	
		n	**%**	***n***	**%**	***n***	**%**	***p*-value***
Country of origin	Spain	99	88.4%	94	90.4%	49	72.1%	0.013
	Foreign	13	11.6%	10	9.6%	19	27.9%	
Newborn sex	Male	57	50.9%	54	51.9%	33	48.5%	0.812

	Female	55	49.1%	50	48.1%	35	51.5%	

		Period	*n*	Mean	*SD*	*p*-value**
	Maternal age	2010-2013	112	32.5	4.6	0.607
		2014-2017	104	33.2	5.9	
		2018-2021	68	33.6	5.6	
	Birth weight	2010-2013	112	2637.3	465.7	0.644
		2014-2017	104	2695.7	466.8	
		2018-2021	68	2631.3	381.2	

Group 9		2010-2013		2014-2017		2018-2021		
		n	*%*	*n*	*%*	*n*	*%*	*p*-value*
Country of origin	Spain	13	72.2%	13	81.3%	13	100.0%	-
	Foreign	5	27.8%	3	18.8%	0	0.0%	
Newborn sex	Male	10	55.6%	6	37.5%	3	23.1%	0.367
	Female	8	44.4%	10	62.5%	10	76.9%	

		Period	*n*	Mean	*SD*	*p*-value**
	Maternal age	2010-2013	18	32.8	5.9	0.727
		2014-2017	16	33.0	5.6	
		2018-2021	13	34.1	5.8	
	Birth weight	2010-2013	18	3502.7	560.5	0.116
		2014-2017	16	3438.1	599.9	
		2018-2021	13	3104.6	420.1	

Group 10		2010-2013		2014-2017		2018-2021		
		n	*%*	*n*	*%*	*n*	*%*	*p*-value*
	Spain	326	81.3%	279	83.0%	176	73.0%	0.023

Country of origin	Foreign	75	18.7%	57	17.0%	65	27.0%	
Newborn sex	Male	238	59.4%	192	57.1%	138	57.3%	0.654
	Female	163	40.6%	144	42.9%	103	42.7%	

	Period	*n*	Mean	*SD*	*p*-value**
Maternal age	2010-2013	401	30.4	5.9	0.041
	2014-2017	336	31.5	6.4	
	2018-2021	241	31.5	6.8	
Birth weight	2010-2013	401	2623.4	489.2	0.107
	2014-2017	336	2580.4	451.1	
	2018-2021	241	2543.1	472.3	

*Chi-square test; **Kruskal Wallis test; RTGCS: Robson Ten Group Classification System; HULR: Hospital Universitario de la Ribera.
